# Comparative gastric microbiota profiles in non-ulcer dyspepsia and peptic ulcer patients

**DOI:** 10.1186/s12866-025-04607-y

**Published:** 2025-12-15

**Authors:** Silva Polat Sari, Aliye Soylu, Kivanc Derya Peker, Gokhan Adas, Ozer Akgul, Burcu Sapmaz, Yasar Ali Oner, Pelin Yuksel Mayda, Reyhan Caliskan

**Affiliations:** 1 Vocational School of Health Services, Istanbul Aydin University, Istanbul, 34295 Turkey; 2 Department of Gastroenterology, Bakirkoy Dr. Sadi Konuk Training and Research Hospital, University of Health Sciences, Istanbul, 34147 Turkey; 3 Department of Perfusion, Faculty of Health Sciences, Istanbul Gelisim University, Istanbul, 34310 Turkey; 4 Department of General Surgery, Prof. Dr. Cemil Tascioglu City Hospital, University of Health Sciences, Istanbul, 34147 Turkey; 5 Department of Medical Microbiology, Faculty of Medicine, Istanbul Health and Technology University, Istanbul, 34445 Turkey; 6 Department of Medical Microbiology, Faculty of Medicine, Istanbul Aydin University, Istanbul, 34295 Turkey; 7 Department of Medical Microbiology, Faculty of Medicine, Uskudar University, Istanbul, 34662 Turkey; 8 Department of Medical Microbiology, Faculty of Medicine, Samsun University, Samsun, 55080 Turkey

**Keywords:** Peptic ulcer disease, Helicobacter pylori, Gastric microbiota, Oral microbiota

## Abstract

**Background:**

Recent evidence suggests that the human stomach hosts a diverse microbiota beyond *Helicobacter pylori*, and that shifts in microbial composition may influence gastric health. In particular, oral-origin bacteria may dominate the gastric niche in the absence of *H. pylori*, yet their specific roles in different gastroduodenal disorders remain unclear. This study aimed to profile and compare the gastric microbiota composition in Turkish patients with non-ulcer dyspepsia (NUD) and peptic ulcer disease (PUD), in order to better understand microbial profiles potentially associated with gastroduodenal disease.

**Methods:**

Ninety-eight patients underwent endoscopic evaluation and were divided into two groups according to the presence or absence of ulcers. Group 1 (*n* = 52) included individuals with NUD, while Group 2 (*n* = 46) comprised patients with PUD. Gastric biopsy samples from both groups were analyzed for the relative abundance of *H. pylori* using quantitative real-time PCR (qPCR), and next-generation sequencing was employed for a comprehensive analysis of the gastric microbiota.

**Results:**

In total, *H. pylori* DNA was detected in 71.4% (70/98) of the samples, with a significantly higher prevalence in PUD patients (82.6%) compared to NUD patients (61.5%) (*p* = 0.02). Distinct microbial profiles were observed based on *H. pylori* status. In NUD patients, *Alloprevotella* showed significantly higher relative abundance in *H. pylori*-negative samples (*p* < 0.05). Among PUD patients, the absence of *H. pylori* was associated with increased levels of *Porphyromonas* and *Neisseria* compared to NUD patients without *H. pylori* (*p* < 0.05). These genera, typically associated with the oral cavity, appeared to expand opportunistically when *H. pylori* was absent.

**Conclusions:**

The absence of *H. pylori* in gastric disorders was linked to a notable shift in microbiota composition, with increased representation of oral-origin bacteria such as *Alloprevotella*, *Porphyromonas*, and *Neisseria*. These findings, observed in a Turkish patient cohort, may reflect a potentially compensatory or opportunistic microbial shift in *H. pylori*-negative gastroduodenal disease. As exploratory findings, this study represents the first analysis from Türkiye comparing gastric microbiota profiles in NUD and PUD patients and provides novel regional insight into gastric microbial ecology.

## Background

Non-ulcer dyspepsia (NUD) and peptic ulcer disease (PUD) are prevalent gastrointestinal disorders that present with varying incidence rates depending on geographic and demographic factors. These conditions significantly impact patients’ quality of life by causing persistent gastric discomfort. NUD is characterized by symptoms such as heartburn, bloating, indigestion, and epigastric pain, which are associated with multiple factors, including stress, dietary habits, medication use, and *H. pylori* infection. Conversely, PUD, though less frequent, remains a clinically significant condition primarily attributed to *H. pylori* infection and prolonged use of non-steroidal anti-inflammatory drugs (NSAIDs) [1].

The identification of *H. pylori* has challenged the long-standing notion of gastric sterility and underscored its pivotal role in the pathogenesis of gastric disorders. This bacterium employs various virulence mechanisms to induce mucosal damage and inflammation, leading to gastritis, peptic ulcers, and in some cases, gastric malignancies. Furthermore, the rise in antibiotic resistance has complicated the eradication of *H. pylori*, making its treatment increasingly challenging [1–3]. Chronic *H. pylori* infection alters the gastric environment by elevating pH levels, thereby promoting the proliferation of non-*H. pylori* microorganisms. Advanced sequencing technologies have demonstrated that the gastric microbiota in healthy individuals primarily comprises five major phyla of bacteria: *Proteobacteria*,* Firmicutes*,* Bacteroidetes*,* Actinobacteria*, and *Fusobacteria* [4, 5].

The composition of gastric microbiota is influenced by several factors, including geographical location, diet, lifestyle, and medication exposure [4, 6]. Studies indicate that the presence of *H. pylori* correlates with reduced microbial diversity, whereas its eradication is associated with the restoration of bacterial diversity and a potential reduction in gastric cancer risk. However, persistent inflammation following *H. pylori* clearance suggests that additional microbial and host factors may contribute to disease progression [7–10].

Emerging evidence suggests a significant interaction between oral and gastric microbiota, further complicating the pathophysiology of gastrointestinal disorders [11, 12]. Despite the growing research interest in the gastric microbiota, the relationship between gastric microbiota and PUD remains inadequately explored, particularly in Turkiye, where no studies have been conducted on this topic. The aim of this study is the characterization of differences in the composition of the gastric microbiota between patients with NUD and PUD. The secondary objective is to investigate the link between these microbial differences and the diseases and to perform a comparative analysis between the two groups.

## Methods

### Study design

Ninety-eight patients with dyspeptic symptoms participated in this prospective comparative study. Participants were selected from individuals undergoing evaluation at the endoscopy units of the gastroenterology clinics at Istanbul Bakirkoy Dr. Sadi Konuk Training and Research Hospital. Patients underwent a comprehensive assessment, including clinical, laboratory, and radiological evaluations by a gastroenterologist, followed by an endoscopic examination for definitive diagnosis. Biopsy samples were collected from the antrum and corpus regions between April 2019 and February 2020, with a preference for analyzing antrum biopsies. Based on endoscopic findings, patients were classified into two groups:

- Group 1 (*n* = 52): NUD-diagnosed patients.
- Group 2 (*n* = 46): PUD-diagnosed patients.

Exclusion criteria included patients younger than 18 years, those with a history of gastric surgery, gall bladder or biliary disease, prior *H. pylori* eradication therapy, use of antibiotics in the past month, recent use of antisecretory agents, bismuth salts or sucralfate within the past 2 weeks, and those with bleeding or clotting disorders. Ethical approval for the study was obtained from the Ethics Committee of Istanbul Aydın University (Decision No. 2019/82), and informed consent was obtained from all participants.

### Genomic DNA isolation

For DNA isolation, 500 µL of lysis buffer (0.5 µg/µL Proteinase K, 5% Tween^®^ 20, 3 M Guanidinium thiocyanate, 20 mM Tris-HCl, pH 8.0) was added to 200 mg of biopsy tissue. Samples were incubated at 70 °C for 15 min, followed by heat inactivation at 95 °C for 5 min. After adding 500 µL of isopropanol, samples were processed using silica columns with centrifugation. Bound DNA was washed twice with a buffer containing 20 mM NaCl, 2 mM Tris-HCl (pH 7.5), and 80% ethanol. Finally, DNA was eluted in 50 µL of 100 mM Tris-HCl (pH 8.0) and stored at −20 °C until further analysis.

### Molecular Screening of *H. pylori *using quantitative real-time PCR (qPCR)

The presence of *H. pylori* was determined using qPCR. Reactions were set up with 1 ng of template DNA, 1U Hot-Start Taq DNA Polymerase, 0.5 µM of each primer, and 2.5 µM MgCl2. The amplification protocol included an initial denaturation at 95 °C for 3 min, followed by 40 cycles of 95 °C for 10 s and 60 °C for 30 s. The forward (5′-GCTCTCACTTCCATAGGCTATAATGTG-3′) and reverse (5′-GCGCATGTCTTCGGTTAAAAA-3′) primers, along with the probe (FAM-5′-TAGGGCCTATGCCTACCCCTGCGA-3′-BHQ1), targeted the *ureA* gene of *H. pylori* [13]. qPCR was conducted on a LightCycler^®^ 96 Real-Time PCR System (Roche, USA), and the relative abundance of *H. pylori* was calculated using the ΔΔCq method [14].

### High-Resolution melting (HRM) analysis

HRM analysis was carried out before microbiota profiling. The 16 S rRNA gene was amplified using the Bact342f (5’-CCTACGGGAGGCAGCAG-3’) and Bact534r (5’-ATTACCGCGGCTGCTGG-3’) primers, targeting the V4 variable region [15]. The cycling conditions of the PCR included an initial denaturation at 95 °C for 3 min, followed by 40 cycles of 95 °C for 20 s, 53 °C for 20 s and 72 °C for 30 s. Reactions were carried out using HRM Master Mix (Bioeksen Ltd. Co., Turkiye) and analyzed on a Biorad CFX-96 qPCR instrument (Bio-Rad Inc., USA). Each reaction mixture consisted of 1.5 mM MgCl₂, 0.2 mM dNTPs, 1× reaction buffer, 0.1 U recombinant *Taq* DNA polymerase, 1× EvaGreen, 5 ng of DNA template, and 0.5 µM of each primer. The HRM analysis involved gradually increasing the temperature from 60 °C to 95 °C at a rate of 0.05 °C/s while continuously measuring fluorescence. Data normalization and clustering were conducted using HRM Analysis Software, and statistical comparisons were performed using Minitab 17 (Minitab Inc., UK) [16]. Principal component analysis (PCA) of HRM melting profiles was performed to identify microbial similarity clusters among gastric biopsy samples. Based on these PCA-derived clusters, representative DNA pools were generated by combining equal DNA volumes from individual samples within each cluster. These pooled DNA samples were subsequently used for 16 S rRNA gene sequencing to characterize the microbial composition of distinct gastric community profiles [17, 18].

### Microbial community profiling via 16 S rRNA sequencing and bioinformatics analysis

Extracted DNA was subjected to amplicon sequencing of the V3-V4 hypervariable regions of the 16 S rRNA gene using the 515 F (5’-CCTACGGGNGGCWGCAG-3’) and 926R (5’-GACTACHVGGGTATCTAATCC-3’) primers [19]. Libraries were prepared, purified, and quantified before sequencing on an Illumina MiSeq platform (Illumina, USA) with 300 bp paired-end chemistry. Demultiplexing and adapter trimming were performed using CASAVA software (Illumina, USA). Reads with barcode mismatches or sequencing errors were discarded. The cutadapt plugin within QIIME2 was used to remove primer sequences, followed by quality filtering (vsearch join-pairs and quality-filter q-score-joined) and denoising via deblur (deblur denoise-16 S). Taxonomic identification of amplicon sequence variants (ASVs) was applied using the SILVA v132 database and the QIIME2 ‘feature-classifier classify-sklearn’ plugin.

### Statistical analysis

SPSS 25.0 software was used for statistical analyses. The Kolmogorov-Smirnov and Shapiro-Wilk tests were used to assess normality for continuous variables. Descriptive statistics were presented as medians, while categorical variables were expressed as frequencies and percentages. Relation between categorical variables were assessed using the chi-square test. The Mann-Whitney U test was used to compare two independent groups when data did not meet normality assumptions. A p-value < 0.05 was considered statistically significant. For comparisons where statistical significance was observed (*p* < 0.05), bacterial taxa with an average relative abundance of less than 1% were excluded.

## Results

Among the 52 patients diagnosed with NUD, 34.6% were male and 65.4% were female, whereas in the 46 PUD patients, males and females were equally distributed (50%). The mean age was significantly higher in PUD patients (46 ± 14.2 years) compared to NUD patients (38.9 ± 14.5 years) (*p* = 0.037). The distribution of gender did not differ significantly between the groups (*p* > 0.05).

In total, *H. pylori* DNA was detected in 71.4% (70/98) of the samples, with a significantly higher prevalence in PUD patients (82.6%) compared to NUD patients (61.5%) (*p* = 0.02). HRM analysis generated normalized melting curve profiles, and a dendrogram was constructed showing 11 HRM similarity clusters sharing ≥ 95% similarity, encompassing 9 phyla, 63 families, and 119 genera. The microbial profiles of NUD and PUD patients were then compared based on the identified taxonomic groups. Taxonomic and PCA-based analyses revealed compositional differences between the two groups, consistent with the HRM clustering results and relative abundance variations. In NUD patients, the dominant phyla were *Firmicutes* (51.6%) and *Bacteroidetes* (17.3%), whereas in PUD patients, *Firmicutes* (46.5%) and *Epsilonbacteraeota* (23.1%) were predominant. The most abundant genera in both groups were *Streptococcus* (41.5% in NUD, 37% in PUD) and *Helicobacter* (13.3% in NUD, 23% in PUD). The relative abundance of *Helicobacter* and *Neisseria* differed significantly between NUD and PUD patients (*p* < 0.05) (Table 1).

**Table 1:** Microbial abundance rates at phylum and genus level in NUD† and PUD‡ groups

Phylum-genus	NUD† (*n*:46)	PUD‡ (*n*:52)	*p*
*Firmicutes*	51.6%	46.5%	> 0,05
*Streptococcus*	41.5%	37%	> 0,05
*Veillonella*	4%	4%	> 0,05
*Bacteroidetes*	17.3%	18.4%	> 0,05
*Prevotella*	2.2%	2.3%	> 0,05
*Prevotella_7*	8.6%	9%	> 0,05
*Porphyromonas*	3%	3.2%	> 0,05
*Alloprevotella*	2%	1.9%	> 0,05
*Proteobacteria*	15.3%	9.6%	> 0,05
*Esch. Shigella*	8.2%	0.2%	> 0,05
*Neisseria*	4.2%	5.3%	< 0,05*
*Haemophilus*	0.7%	1.1%	> 0,05
*Ralstonia*	0.4%	1.2%	> 0,05
*Epsilonbacteraeota*	13.5%	23.1%	> 0,05
*Helicobacter*	13.3%	23%	< 0,05*
*Actinobacteria*	1.3%	1.5%	> 0,05

†Non ulcer dispepsia

‡Peptic ulcer disease

*p< 0,05

Analysis indicated that *Epsilonbacteraeota* was significantly overrepresented in *H. pylori*-positive samples, whereas *Bacteroidetes* was more prevalent in *H. pylori*-negative samples (*p* < 0.05). In addition, *Helicobacter* was significantly overrepresented in *H. pylori*-positive samples, while *Prevotella* was more prevalent in *H. pylori*-negative samples (*p* < 0.05) (Table 2).

**Table 2:** Microbiota profile of *H. pylori* positive and negative specimens at phylum and genus level

Phylum-genus	H. pylori + (*n* = 70)	H. pylori - (*n* = 28)	*p*
*Actinobacteria*	1,3%	1,6%	> 0,05
*Bacteroidetes*	16,1%	23,4%	< 0,05*
*Prevotella*	2%	3,1%	< 0,05*
*Prevotella_7*	8,1%	10,6%	> 0,05
*Porphyromonas*	3%	3,4%	> 0,05
*Alloprevotella*	1,7%	2,5%	< 0,05*
*Epsilonbacteraeota*	22,7%	5,2%	< 0,05*
*Helicobacter*	22,8%	5,7%	< 0,05*
*Firmicutes*	47,3%	54%	> 0,05
*Streptococcus*	37,6%	43,9%	> 0,05
*Veillonella*	4%	4%	> 0,05
*Proteobacteria*	11,8%	14,6%	> 0,05
*Esch. Shigella*	3,8%	6,2%	> 0,05
*Neisseria*	4,7%	5%	> 0,05
*Haemophilus*	1%	1%	> 0,05
*Ralstonia*	1%	1%	> 0,05

*p< 0,05

Within the NUD group, regardless of *H. pylori* status, *Firmicutes* remained the dominant phylum (50.6% in *H. pylori*-positive and 53.3% in *H. pylori*-negative patients), and *Streptococcus* was the most prevalent genus (39.9% and 43.9%, respectively). Significant differences were noted between *H. pylori*-positive and negative NUD patients at both the phylum level (*Bacteroidetes*, *Epsilonbacteraeota*) and the genus level (*Alloprevotella*) (*p* < 0.05) (Tables 3 and 4).

**Table 3:** Phylum-level microbiota profile of *H.pylori* positive and negative samples in the NUD**†** and PUD**‡** groups

Phylum	H. pylori + NUD† (*n*:32)	H. pylori – NUD† (*n*:20)	*p*	H. pylori + PUD‡ (*n*:38)	H. pylori – PUD‡ (*n*:8)	*p*
*Actinobacteria*	1,2%	1,4%	> 0,05	1,5%	2%	> 0,05
*Bacteroidetes*	14,1%	22,4%	< 0,05*	17,7%	25,7%	> 0,05
*Epsilonbacteraeota*	18,1%	6%	< 0,05*	26,5%	3,2%	< 0,05*
*Firmicutes*	50,6%	53,3%	> 0,05	44,5%	55,4%	> 0,05
*Proteobacteria*	15,1%	15,6%	> 0,05	8,9%	12,3%	> 0,05

†Non ulcer dispepsia

‡Peptic ulcer disease

*p< 0,05

**Table 4:** Genus-level microbiota profile of *H.pylori* positive and negative samples in the NUD**†** and PUD**‡** groups

Genus	H. pylori + NUD† (*n* = 32)	H. pylori - NUD† (*n* = 20)	*p*	H. pylori + PUD‡ (*n* = 38)	H. pylori – PUD‡ (*n* = 8)	*p*
*Porphyromonas*	3,2%	2,7%	> 0,05	2,8%	5%	< 0,05*
*Alloprevotella*	1,6%	2,5%	< 0,05*	1,7%	2,6%	< 0,05*
*Prevotella*	2%	2,9%	> 0,05	2,1%	3,5%	> 0,05
*Prevotella_7*	7,6%	10,2%	> 0,05	8,5%	11,6%	> 0,05
*Helicobacter*	17,5%	6,7%	> 0,05	27,2%	3,1%	< 0,05*
*Streptococcus*	39,9%	43,9%	> 0,05	35,7%	43,8%	> 0,05
*Veilonella*	4,1%	4,1%	> 0,05	4%	3,8%	> 0,05
*Neisseria*	4,4%	4%	> 0,05	4,9%	7,7%	> 0,05
*Haemophilus*	< 1%	< 1%		1%	1,4%	> 0,05
*Ralstonia*	< 1%	< 1%		1,2%	1,1%	> 0,05
*Esch. Shigella*	8%	8,6%	> 0,05	< 1%	< 1%	

†Non ulcer dispepsia

**Peptic ulcer disease

*p< 0,05

Similarly, in the PUD group, *Firmicutes* was the dominant phylum regardless of *H. pylori* status (44.5% in *H. pylori*-positive and 55.4% in *H. pylori*-negative patients), with *Streptococcus* as the most abundant genus (35.7% and 43.8%, respectively). Significant differences were observed in *Epsilonbacteraeota* abundance between *H. pylori*-positive and negative PUD patients, along with significant variations in *Porphyromonas*,* Alloprevotella*, and *Helicobacter* abundance (*p* < 0.05) (Tables 3 and 4).

Interestingly, in the *H. pylori*-positive NUD group, *Haemophilus* and *Ralstonia*—which were below 1% in NUD—showed a marked increase in the *H. pylori*-positive PUD group. When comparing *H. pylori*-negative NUD and PUD groups, *Neisseria* and *Porphyromonas* were significantly more abundant in PUD patients (*p* < 0.05) (Table 5).

**Table 5:** Microbiota profile between NUD**†** and PUD**‡** groups according to *H. pylori* status at the genus level

Genus	H. pylori + NUD† (*n* = 32)	H. pylori + PUD‡ (*n* = 38)	*p*	H. pylori - NUD† (*n* = 20)	H. pylori - PUD‡ (*n* = 8)	*p*
* Porphyromonas *	3,2%	2,8%	> 0,05	2,7%	5%	< 0,05*
* Alloprevotella *	1,6%	1,7%	> 0,05	2,5%	2,6%	> 0,05
* Prevotella *	2%	2,1%	> 0,05	2,9%	3,5%	> 0,05
*Prevotella_7*	7,6%	8,5%	> 0,05	10,2%	11,6%	> 0,05
*Helicobacter*	17,5%	27,2%	> 0,05	6,7%	3,1%	> 0,05
*Streptococcus*	39,9%	35,7%	> 0,05	43,9%	43,8%	> 0,05
*Veilonella*	4,1%	4%	> 0,05	4,1%	3,8%	> 0,05
*Neisseria*	4,4%	4,9%	> 0,05	4%	7,7%	< 0,05*
*Haemophilus*	0,008%	1%	< 0,05*	0,007%	1,4%	> 0,05
*Ralstonia*	0,004%	1,2%	< 0,001**	0,005%	1,1%	> 0,05
*Esch. Shigella*	8%	0,002%	> 0,05	8,6%	0,001%	> 0,05

†Non ulcer dispepsia

‡Peptic ulcer disease

*p< 0,05 **p< 0,001

## Discussion

NUD and PUD are common gastric disorders worldwide. *H. pylori*, which was classified as a Group 1 human carcinogen by the World Health Organisation (WHO) in 1994, is strongly associated with a variety of gastrointestinal diseases, including NUD, chronic gastritis, PUD and gastric cancer [1, 20]. Advances in next-generation sequencing and bioinformatics have significantly improved our understanding of the relationship between *H. pylori* and gastrointestinal diseases, leading to an increasing focus on gastric microbiota research. The prevalence of *H. pylori* infection in adults varies globally, ranging from 11.5% to 95.5%, depending on geographic and socioeconomic factors. In Turkiye, the reported prevalence is between 71% and 83% [21]. In the present study, *H. pylori* DNA was detected in 70 out of 98 samples (71.4%). Literature indicates that *H. pylori* prevalence in NUD patients ranges from 31.2% to 65% [22]. Our findings, showing a positivity rate of 61.5% among NUD patients, are consistent with these reports. The relation between *H. pylori* and PUD is well established, with *H. pylori* infection found in approximately 90% of duodenal ulcers and 70–90% of gastric ulcers. In our study, *H. pylori* was detected in 82.6% of PUD patients. Variations in *H. pylori* prevalence may be attributed to differences in geographic distribution, ethnicity, age, gender, and other demographic factors [4, 6].

Healthy population studies have identified *Firmicutes*, *Bacteroidetes*, *Proteobacteria*, *Actinobacteria* and *Fusobacteria* as a dominant bacterial phyla in the gastric microbiota [4, 5]. Among the most commonly detected genera are *Streptococcus*, *Prevotella*, *Fusobacterium*, *Veillonella*, *Neisseria*, and *Haemophilus*. Recent research has also emphasized the role of the oral microbiota, which comprises over 700 species and represents the second most diverse microbiome after the intestinal microbiota [23]. *Streptococcus* constitutes a significant proportion of the oral microbiota (12–66%), while other genera are present in lower abundances (6–29%) [24]. The translocation of oral bacteria into the gastric environment can contribute to inflammation through the production of toxic metabolites, potentially increasing the risk of gastric malignancies [25].

Several studies suggest that gastric microbiota composition differs between NUD patients and healthy individuals. An increased abundance of the *Firmicutes* phylum and *Streptococcus* genus in NUD patients has been correlated with symptom severity [26]. Furthermore, *Cutibacterium acnes* (formerly *Propionibacterium acnes*) has been reported to be more prevalent in NUD patients compared to PUD patients, suggesting a possible role in dyspepsia alongside *H. pylori* [27]. Additionally, Igarashi et al. observed a predominance of *Bacteroidetes* over *Proteobacteria* in NUD patients compared to healthy individuals, further supporting the hypothesis of microbiota alterations in dyspeptic conditions [28].

In this study, *Firmicutes* (51.6%) and *Bacteroidetes* (17.3%) were identified as the dominant phyla in the NUD group, whereas *Firmicutes* (46.5%) and *Epsilonbacteraeota* (23.1%) were predominant in the PUD group. The most frequently detected genera were *Streptococcus* and *Helicobacter*. In particular, the relative abundances of *Helicobacter* and *Neisseria* were significantly overrepresented in the PUD group than in the NUD group (*p* < 0.05) (Table 1). These findings suggest that *Neisseria*, in conjunction with *Helicobacter*, may play a role in ulcer pathogenesis.

Several studies have investigated how *H. pylori* infection affects gastric microbial diversity and composition [7–10]. In this study, comparing microbiota profiles between *H. pylori* positive and negative samples, *Epsilonbacteraeota* significantly dominated in *H. pylori* presence, whereas *Bacteroidetes* significantly increased in absence (*p* < 0.05). At the genus level, *Helicobacter* was significantly enriched in *H. pylori*-positive samples, while *Alloprevotella* and *Prevotella* were significantly overrepresented in *H. pylori*-negative samples (*p* < 0.05) (Table 2). These findings suggest that the presence/absence of *H. pylori* may influence the composition of specific bacterial taxa within the gastric microbiota, which may affect overall microbial diversity.

In this study, the dominant phyla in the *H. pylori*-positive NUD group were *Firmicutes* (50.6%) and *Epsilonbacteraeota* (18.1%), whereas in the *H. pylori*-negative group, *Firmicutes* (53.3%) and *Bacteroidetes* (22.4%) were predominant (Table 3). At the genus level, *Streptococcus* (39.9%) and *Helicobacter* (17.5%) were the most abundant in the *H. pylori*-positive group, while *Streptococcus* (43.9%) and *Prevotella_7* (10.2%) were the most prevalent in the *H. pylori*-negative group. Notably, *Alloprevotella* was significantly overrepresented in the *H. pylori*-negative group (*p* < 0.05). Although the increase in *Streptococcus* and *Prevotella_7* proportions in the *H. pylori*-negative NUD group was not statistically significant in this study (Table 4), previous research has suggested that an elevated abundance of *Streptococcus* in *H. pylori*-negative individuals may be associated with gastroduodenal diseases [29]. Furthermore, it has been reported that proton pump inhibitor (PPI) use can lead to an increased abundance of these bacteria [30].

Research on the gastric microbiota in PUD remains limited. Studies indicate that *Firmicutes* are the predominant phylum in both healthy individuals and PUD patients, whereas *Proteobacteria* are more prevalent in cases with mucosal erosion. The key genera associated with PUD include *Ruminococcus_2*,* Agathobacter*,* Alistipes*,* Helicobacter*,* Bacteroides*, and *Faecalibacterium* [31]. *Prevotella*,* Neisseria* and *Streptococcus* have also been identified in both *H. pylori*-positive and negative gastric mucosa [32–34]. One study by Hu et al., using mass spectrometric analysis, reported that *Streptococcus* and *Neisseria* were the dominant genera in gastric mucosal tissue tested positive for *H. pylori* [35]. Furthermore, microbial diversity in the gastric mucosa of *H. pylori*-positive duodenal ulcer patients has been found to be higher compared to those with gastric antrum ulcers [32]. Notably, gastric ulcers are associated with a higher oncogenic potential than duodenal ulcers, and previous research suggests a link between reduced microbial diversity and oncogenesis [36].

In this study, the dominant phyla in *H. pylori*-positive PUD patients were *Firmicutes* (44.5%) and *Epsilonbacteraeota* (26.5%), whereas in the *H. pylori*-negative group, *Firmicutes* (55.4%) and *Bacteroidetes* (25.7%) were predominant (Table 3). At the genus level, *Streptococcus* (35.7%) and *Helicobacter* (27.2%) were the most abundant in *H. pylori*-positive PUD patients, while *Streptococcus* (43.8%) and *Prevotella_7* (11.6%) were the most prevalent in the *H. pylori*-negative group (Table 4). Additionally, *Porphyromonas* and *Alloprevotella* were significantly overrepresented in *H. pylori*-negative PUD patients (Table 4). These findings suggest that certain taxa within the oral microbiota, including *Alloprevotella*,* Porphyromonas*, and *Streptococcus*, may play a role in the pathogenesis of peptic ulcer disease.

Oral microbiota dysbiosis has been associated with both gastrointestinal and dental disorders.^11,23^ Studies suggest that certain oral microorganisms, including *Clostridium*,* Porphyromonas*, and *Prevotella*, may contribute to oncogene activation and inflammatory processes [37, 38]. Among the microbial taxa frequently linked to gastric cancer are oral commensals and opportunistic pathogens such as *Neisseria*, *Alloprevotella*, and *Aggregatibacter* [39]. It has been reported that oropharyngeal commensals, including *Streptococcus*,* Bifidobacterium*,* Escherichia*,* Pseudomonas*,* Neisseria*,* Staphylococcus*,* Veillonella*,* Klebsiella*,* Alloprevotella*,* Aggregatibacter*,* Porphyromonas endodontalis*,* Bacillus*,* Haemophilus*, are dominant in gastric cancer patients [40, 41]. In this study, a significant increase in *Haemophilus* and *Ralstonia* was observed in the PUD group compared to the NUD group, where their relative abundance was below 1% (*p* < 0.05) (Table 5). Notably, *Ralstonia* has been recognized as an opportunistic pathogen in immunocompromised individuals and may contribute to inflammatory responses [42, 43]. Furthermore, a comparison between *H. pylori*-negative NUD and PUD groups revealed that *Porphyromonas* and *Neisseria* were significantly overrepresented in the PUD group (*p* < 0.05) (Table 5). These results indicate that the oral microbiota may be a significant contributor to the risk of developing gastroduodenal diseases.

In our study, low levels of *Helicobacter* were found even in *H. pylori*-negative groups (Tables 2, 4 and 5). This finding may suggest the possible presence of other *Helicobacter* species [44].

Certain bacterial species, particularly those other than *H. pylori*, have been reported to contribute to nitrite reduction, enhance inflammatory responses, and increase the risk of gastric cancer [25, 45]. In acidic conditions, nitrites and nitrates react with amines, leading to the formation of carcinogenic nitrosamines, which are present in processed meats, alcohol, and cigarette smoke. These compounds can exacerbate gastric mucosal inflammation and accelerate the progression of precancerous lesions [45–48].

Bacteria capable of nitrosation, including *Escherichia coli*,* Streptococcus*,* Nitrospirae*,* Veillonella*,* Haemophilus*,* Staphylococcus*,* Clostridium*, and *Neisseria*, can generate carcinogenic N-nitroso compounds (NOCs), thereby increasing gastric cancer risk [39, 48, 49]. Previous reports have shown that the relative abundance of non-*H. pylori* gastric bacteria involved in nitrate reduction and nitrosation is higher in gastric cancer patients than controls [48, 50]. Although this study did not include a gastric cancer cohort, it is noteworthy that the genus *Neisseria* was significantly overrepresented in *H. pylori*-negative PUD patients in comparison to *H. pylori*-negative dyspepsia patients (Table 5). The enrichment of oral-origin taxa such as *Neisseria*, *Porphyromonas*, and *Alloprevotella* in *H. pylori*–negative PUD patients may have important clinical relevance. These bacteria have been associated with proinflammatory processes and nitrosation pathways, suggesting that they could contribute to mucosal damage independently of *H. pylori* [25, 51]. Although our findings are exploratory, they raise the possibility that oral-origin bacteria may serve as biomarkers for identifying high-risk patients or for informing the development of dyspepsia management strategies. However, the age imbalance between the groups may have contributed to the microbial differences observed. Although the potential confounding effect of age cannot be entirely excluded, the results should be interpreted in light of the exploratory nature of the study. Moreover, while patients with recent antibiotic or PPI use were excluded, the broader effects of long-term or past medication use on gastric microbiota cannot be completely ruled out [52–54]. Future research should further evaluate whether these taxa might be integrated into diagnostic or prognostic frameworks for gastroduodenal diseases.

This study has several limitations. First, the absence of a gastric cancer group limited the ability to compare the findings with malignant conditions. Moreover, given the exploratory nature of this study, 16 S rRNA sequencing was performed on pooled DNA samples derived from HRM-defined similarity clusters rather than on individual biopsies. This approach was adopted to maximize the representativeness of microbial community profiles while maintaining cost-effectiveness and methodological feasibility. The HRM-based clustering and representative pooling method has been shown to be reliable and reproducible in various studies [17, 18]. As HRM provides melting-based clustering rather than sequence-level data, classical alpha and beta diversity analyses could not be applied. Instead, relative abundance distributions and PCA-based similarity mapping were used to describe group-level compositional trends. Furthermore, because sequencing was performed on pooled DNA samples rather than on individual biopsies, individual-level variance could not be estimated, and therefore measures of dispersion were not included in the results. This approach provides an initial and valuable insight into gastric microbial ecology and adds novel regional insight, forming a methodological basis for future sequencing-based investigations. In addition, the relatively small number of patients in certain subgroups, particularly *H. pylori*–negative PUD, may have reduced the statistical power of these comparisons. This limitation likely reflects the low prevalence of *H. pylori*–negative PUD in the study population. Nevertheless, these exploratory subgroup analyses provide preliminary insights into the microbial features of this clinically relevant but underrepresented group. Furthermore, as multiple comparisons were conducted across numerous bacterial taxa, the absence of correction for multiple testing may have increased the risk of type I error. However, given the exploratory nature and limited sample size of this study, statistical corrections were not applied to avoid potential loss of biologically relevant signals. Additionally, differences in age distribution and the lack of detailed data on long-term medication history represent potential confounders that may have affected the gastric microbiota composition. Moreover, dietary habits, oral health status, and socioeconomic factors were not evaluated in this study. These unmeasured variables may have influenced microbial composition and should be taken into account when interpreting the results. In addition, the absence of parallel oral microbiota data limited a more detailed exploration of potential interactions between gastric and oral bacteria. Future studies incorporating both oral and gastric sampling may better elucidate these complex microbial relationships.These limitations should be acknowledged and considered when interpreting the findings.

Despite these limitations, our study represents the first investigation in Türkiye to compare the gastric microbiota profiles of NUD and PUD patients. The findings contribute novel regional data on gastric microbial composition and provide a valuable starting point for future large-scale metagenomic studies based on individual biopsies, including gastric cancer patients, and incorporating more comprehensive statistical approaches. Overall, these exploratory findings highlight the need for future studies with larger and more balanced cohorts, incorporating more comprehensive statistical models to confirm and expand upon our observations.

Furthermore, these findings highlight the potential clinical relevance of non-*H. pylori* gastric microbiota in gastroduodenal diseases. Identifying specific microbial taxa associated with *H. pylori*–negative conditions may support the development of novel diagnostic and preventive approaches and contribute to a better understanding of host–microbe interactions in gastric pathology. Despite its exploratory nature, this study provides regionally relevant data that may inform future microbiome-based clinical research. Understanding these patterns may help to refine clinical perspectives on the role of gastric microbiota in non-*H. pylori* gastroduodenal disorders.

## Conclusion

Although numerous studies have explored the relationship between non-*H. pylori* gastric microbiota and gastric cancer, research focusing on the microbiota composition in PUD patients remains limited. This study represents the first investigation from Türkiye comparing the gastric microbiota profiles of NUD and PUD patients. Our findings revealed a significant enrichment of *Porphyromonas*, *Neisseria*, and *Alloprevotella* in *H. pylori*-negative PUD cases. This observation may be associated with the possible influence of oral-origin microorganisms on gastric microbial balance and disease susceptibility.

As an exploratory first step, this study provides novel regional insight into gastric microbial ecology and establishes a framework for future research. Larger-scale metagenomic and functional analyses with more diverse cohorts across different gastric disease phenotypes will contribute to a more comprehensive understanding of microbial interactions and ecological dynamics. Such efforts will enable the development of a deeper understanding of the complex relationship between gastric and oral microbiota.

## Data Availability

Sequence data that support the findings of this study have been deposited in the NCBI Sequence Read Archive (SRA) under BioProject ID PRJNA1297040.

## Abbreviations

NUD: Non ulcer dyspepsia
PUD: Peptic ulcer disease
qPCR: quantitative real-time PCR
NSAID: Non-steroidal anti-inflammatory drugs
HRM: High Resolution Melting
NOCs: N-nitroso compounds
